# A Comparison of Phenotypic Traits Related to Trypanotolerance in Five West African Cattle Breeds Highlights the Value of Shorthorn Taurine Breeds

**DOI:** 10.1371/journal.pone.0126498

**Published:** 2015-05-08

**Authors:** David Berthier, Moana Peylhard, Guiguigbaza-Kossigan Dayo, Laurence Flori, Souleymane Sylla, Seydou Bolly, Hassane Sakande, Isabelle Chantal, Sophie Thevenon

**Affiliations:** 1 CIRAD, UMR INTERTRYP, F-34398, Montpellier, France; 2 CIRDES, 01 BP 454 Bobo-Dioulasso 01, Burkina Faso; 3 INRA, UMR 1313 GABI, F-78350, Jouy-en-Josas, France

## Abstract

**Background:**

Animal African Trypanosomosis particularly affects cattle and dramatically impairs livestock development in sub-Saharan Africa. African Zebu (AFZ) or European taurine breeds usually die of the disease in the absence of treatment, whereas West African taurine breeds (AFT), considered trypanotolerant, are able to control the pathogenic effects of trypanosomosis. Up to now, only one AFT breed, the longhorn N’Dama (NDA), has been largely studied and is considered as the reference trypanotolerant breed. Shorthorn taurine trypanotolerance has never been properly assessed and compared to NDA and AFZ breeds.

**Methodology/Principal Findings:**

This study compared the trypanotolerant/susceptible phenotype of five West African local breeds that differ in their demographic history. Thirty-six individuals belonging to the longhorn taurine NDA breed, two shorthorn taurine Lagune (LAG) and Baoulé (BAO) breeds, the Zebu Fulani (ZFU) and the Borgou (BOR), an admixed breed between AFT and AFZ, were infected by *Trypanosoma congolense* IL1180. All the cattle were genetically characterized using dense SNP markers, and parameters linked to parasitaemia, anaemia and leukocytes were analysed using synthetic variables and mixed models. We showed that LAG, followed by NDA and BAO, displayed the best control of anaemia. ZFU showed the greatest anaemia and the BOR breed had an intermediate value, as expected from its admixed origin. Large differences in leukocyte counts were also observed, with higher leukocytosis for AFT. Nevertheless, no differences in parasitaemia were found, except a tendency to take longer to display detectable parasites in ZFU.

**Conclusions:**

We demonstrated that LAG and BAO are as trypanotolerant as NDA. This study highlights the value of shorthorn taurine breeds, which display strong local adaptation to trypanosomosis. Thanks to further analyses based on comparisons of the genome or transcriptome of the breeds, these results open up the way for better knowledge of host-pathogen interactions and, furthermore, for identifying key biological pathways.

## Introduction

Animal African Trypanosomosis (AAT), a neglected tropical disease, particularly affects cattle and dramatically impairs the development of livestock production systems in sub-Saharan Africa [1], [2], [3]. AAT is caused by trypanosomes, blood-borne protozoan parasites from the *Trypanosoma* genus transmitted by haematophagous insects, generally tsetse flies (*Glossina* genus) [4]. According to the Food and Agriculture Organization, they are responsible for the death of 3 million cattle per year, with an annual economic loss estimated at $1–1.2 billion [5], [6]. No vaccine is available and African livestock breeders annually administer about 35 million curative and preventive treatments, against which certain trypanosome strains have become resistant [7]. Although eradication of tsetse flies has proved possible in a few particular cases [8], it remains impossible in most regions, where integrated control strategies are needed [9].

In this context where AAT remains a dramatic constraint to livestock production, the investigation and optimum utilization of animal genetic resources adapted to harsh environments [10] paves the way for improving AAT control. Indeed, African Zebu breeds (AFZ), as well as European taurine breeds (EUT), are highly susceptible to the disease caused by three tsetse-transmitted trypanosome species, *Trypanosoma congolense*, *T*. *vivax* and *T*. *brucei brucei* [11]. Accordingly, breeding susceptible animals is extremely difficult in highly enzootic areas, even when trypanocide drugs are used. Conversely, West Africa taurine breeds (AFT), which are found in humid and sub-humid areas of West Africa where AAT is endemic [12], are considered as able to tolerate the disease, i.e., able to reduce parasitaemia, control anaemia, and remain productive in these areas [13], [14], [15]. West African cattle trypanotolerance would seem to be an adaptive character in response to selective pressure due to trypanosomes. Indeed, the introduction of taurine cattle in West Africa pre-dated the arrival of indicine breeds by at least 3,000 years [16]. However, the trypanotolerant status of AFT in comparison with some AFZ has only been clearly demonstrated under field conditions [17], [18], and during experimental infections [14], [19], [20], [21], [22], [23] for the longhorn taurine N’Dama breed (NDA), which was thus considered as the trypanotolerant reference breed. Conversely, experimental data to assess the degree of trypanotolerance in shorthorn taurine, including in particular Baoulé (BAO), Somba (SOM) and Lagune (LAG), are lacking [24]. The few studies concerning the Baoulé breed under field challenge with *Trypanosoma* sp [25], [26], [27], had the drawback of combining the variability of the host, parasite (at species and strain levels) and tsetse bites, which resulted in a large variability of responses in BAO cattle. Moreover, support for the trypanotolerance of the Lagune breed is based solely on its agro-ecology [24]. A single study based on an experimental infection compared LAG and Borgou (BOR) breeds [28], the latter being a stabilized admixed breed between AFT and AFZ representative of the West African bovine hybrid zone [29], [30], but the study lacked a proper susceptible control breed and used a trypanosome species, *T*. *brucei brucei*, which is usually less pathogenic than *T*. *vivax* and *T*. *congolense* [11].

In this context of the absence of a proper comparison of trypanotolerant traits between the main West African cattle breeds, we sought here to compare for the first time the trypanotolerant/susceptible phenotype of five local West African breeds (LAG, BAO, NDA, ZFU and BOR) during a single experimental infection with *T*. *congolense*, considered as the main pathogenic trypanosome species for cattle [11], [31], [32]. All the monitored cattle were well characterized at their genome level and the usual parameters, such as parasitaemia, packed cell volume (PCV), and leukocyte counts were analysed using longitudinal data.

## Methods

### Ethics statement

The experimental protocol was reviewed and approved by the Burkinabe Ethics Committee (Project no. A002-2013 / CE-CM) and it was carried out by qualified and experienced personnel under the supervision of a veterinarian, according to the guidelines of the World Organisation for Animal Health. For trypanosome production, three mice were deeply anaesthetized by a combination of ketamine (at 200 mg/kg) and xylazine (10mg/kg) followed by exsanguination by intra-cardiac puncture. No cattle were euthanized or died during the challenge experiment. Clinical examination was performed each day and when an animal presented a deterioration of its general body condition or suffered signs of severe anaemia (threshold at 15% PCV), it was treated using diminazene aceturate (Veriben, curative treatment) at 7 mg/kg intramuscular, and Fercobsang. The animal continued to be monitored to check for treatment effectiveness and the increase in haematocrit. In any event, all cattle were treated with diminazene aceturate at 7mg/kg at the end of the experiment (133 days post-infection).

### Experimental animals

**Fig 1:**
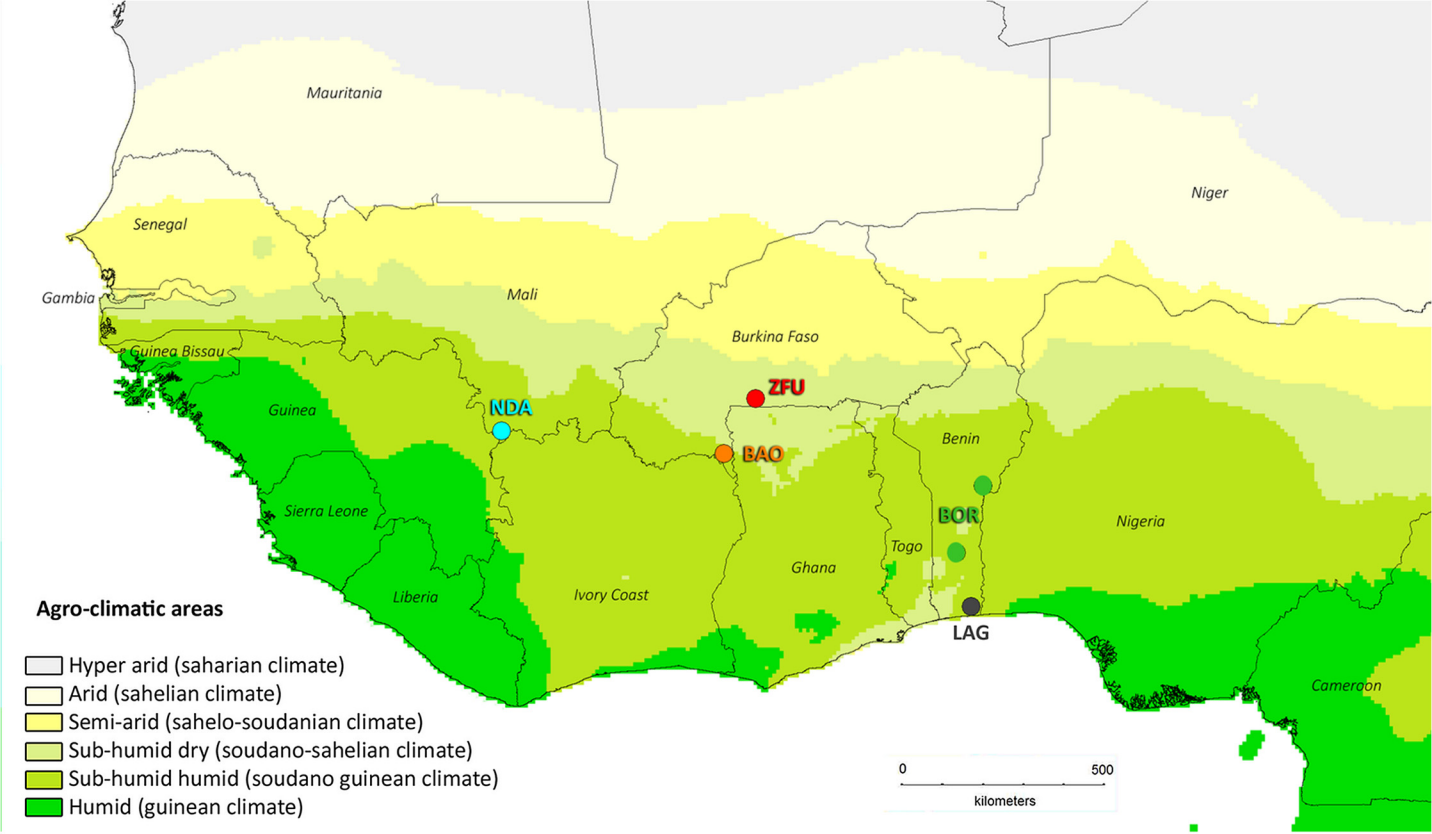
Map representing the geographical origin of the breeds used in the experiment. DIVA-GIS software version 7.5 [35] with the BIOCLIM system [36] was used to obtain the map. The different agro-climatic zones were defined based on the annual precipitation of the Worldclim database (http://www.worldclim.org) and according to the OECD 2009 criteria [37]. According to [38], the current northern limit of tsetse presence is slightly above the limit between the semi-arid and sub-humid dry areas. DIVA-GIS and spatial data are freely available and downloadable from http://www.diva-gis.org/

Cattle from five local West African breeds were used for the experiment: eight Baoulé (BAO), eight Borgou (BOR), seven Lagune (LAG), eight N’Dama (NDA) and eight Sudanese Fulani Zebu (ZFU). Unrelated males aged from one to two years and a half were imported to the experimental station from their locations of origin, namely the Lobi region in Burkina Faso for BAO, the Betecoucou and Okpara stations in Benin for BOR, the Samiondji station in Benin for LAG, the Madina Diassa region in Mali for NDA and the Solenzo region in Burkina Faso for ZFU. Location origins are shown in Fig 1. The inclusion criteria were absence of trypanosomes in cattle blood examined according to the parasitological technique of Murray ([33]) and negative serological responses using indirect IgG (immunoglobulin G) antibody ELISA (Enzyme Linked Immuno-Sorbent Assay) with *T*. *vivax*, *T*. *brucei brucei* and *T*. *congolense* total antigens ([34]), at the time of the veterinary survey.

On their arrival at the CIRDES station, the cattle were kept in a cowshed under mosquito netting, treated with an anthelmintic (Albendazole 2,500 mg Vermitan) *per os*, a combination of ivermectin and clorsulon in SC (0.2 mg and 2mg/kg respectively, Ivomec-D), deltamethrin spray (Vectocid) and a trypanocide drug (diminazene aceturate at 7 mg/kg, Veriben). They were also vaccinated against haemorrhagic septicaemia (Pastovax), contagious bovine pleuropneumonia (Perivax) and blackleg disease (Symptovax). The animals were kept for at least four weeks before being infected.

In order to perform a genetic assignment of each animal to its corresponding breed, the cattle were genotyped using the Illumina BovineSNP50 chip assay v2 [39] at the INRA Labogena platform (Jouy-en-Josas, France) using standard procedures (http://www.illumina.com/products/bovine_snp50_whole-genome_genotyping_kits.ilmn). These new genotypes were combined with those already available [29,39,40]. This dataset included i) 137 AFT individuals from the NDA (ND3), SOM, BAO, and LAG breeds, ii) 138 ZEB individuals from the ZFU, Zebu Bororo (ZBO), Zebu from Madagascar (ZMA), Gir (GIR), Nelore (NEL) and Brahman (BRM) breeds, iii) 152 EUT individuals from the Salers (SAL), Limousin (LMS), Holstein (HOL), Montbéliard (MON), Angus (ANG) and Hereford (HFD) breeds, iv) 24 EUTxZEB admixed individuals from the Santa Gertrudis (SGT) breed, v) 26 EUTxAFT admixed individuals from the Oulmes Zaër (OUL) breed and vi) 68 AFTxZEB admixed individuals from the BOR and Kuri (KUR) breeds. Following the procedure described in [40], [30], SNPs that i) displayed a minor allele frequency <0.01 over the whole sample; ii) were genotyped on <75% of the individuals in at least one breed and iii) did not pass Hardy-Weinberg equilibrium tests, were discarded from the final dataset. A total of 43,845 SNPs genotyped on 545 animals was considered in the analysis. Population structure was analysed through a principal component analysis (PCA), based on individual SNP genotype data with *smartpca* software [41] and visualized using R package *ade4* [42], and through an unsupervised genotype-based hierarchical clustering (UHC) of the individuals using the maximum likelihood method implemented in *Admixture 1*.*04* [43].

### Trypanosome challenge

The trypanosome used for the challenge was *T*. *congolense* IL1180, a double clone derivative of Ser/71/STIB/212 [44], [45]. It was chosen because it was isolated from a lion in Tanzania, and thus no cattle could have been infected by this strain before. It has a medium pathogenicity, allowing the development of a chronic disease [46] and it has been used in previous experiments [21], [22], [47], [48]. The intravenous inoculation route was selected to perfectly control the quantity of injected trypanosomes, which is not possible with tsetse fly inoculation, and in order to avoid cattle immune response interactions with tsetse saliva antigens. Trypanosomes were first multiplied on mice, purified on DEAE cellulose, quantified on Malassez cells and injected at a dose of 10^5^ trypanosomes per head of cattle.

### Monitoring of phenotypic variables

Several variables were monitored before and during experimental infection. First, blood samples from the jugular vein were collected on EDTA anticoagulant (ethylenediamine tetraacetic acid) five times before infection (days: -25, -11, -7, -3 and -1) and then three times/week during infection, to measure PCV and parasitaemia. PCV was read for each animal in a microhaematocrit tube after centrifugation for 5 min at 12,500 rpm. Parasitaemia was scored according to the phase-contrast buffy coat technique [33], [49] and expressed as the number of trypanosomes/ml.

Erythrocyte and haemoglobin rates (RBC and Hb respectively) were measured -7 and -3 days before infection, every five days on average over the first two months of infection and then every two weeks over the last 2.5 months, using standard procedures with the ABX micros 60 (Horiba). Reticulocytes were sought using brilliant cresyl blue (Merck) according to the manufacturer’s instructions. Briefly, 5 μl of cattle blood was stained with 5 μl of saline solution (0.58%) of brilliant cresyl blue (150 mg/l) for 30 min. Blood strains were then performed and examined in immersion after drying with a X100 objective. Reticulocytes were counted among 1,000 RBC. Total white blood cells (TWBC) were counted every four days on average, using direct observation with a Mallassez cell.

Blood in dry tubes was collected once a week to harvest serum for indirect IgG ELISA against total antigens of *T*. *congolense* [34]. Results were expressed as a percentage of positivity (PP) using three positive and negative reference samples, as already described in [50].

### Statistical analyses on synthetic variables

Statistical analyses were carried out with R3.1.0 (R development core team 2007, http://www.r-project.org/).

Initially, we used synthetic traits as proposed in [21] and [23] to assess trypanotolerance.

For PCV, RBC and Hb, pre-infection values were the median values recorded before infection, called PCV_Init, RBC_Init and Hb_Init respectively (see Table 1 for the recapitulation of all synthetic variables). The median value (PCV_Med, RBC_Med, Hb_Med) was the median level recorded over the 133 days post trypanosome inoculation. The lowest value (PCV_Min, RBC_Min, Hb_Min) was the absolute lowest level recorded during the course of infection. The maximum decrease (_MD) was the absolute decrease and was calculated as the lowest value minus the pre-infection value. The slope decrease (_SD) was the linear trend in the PCV, RBC or Hb drop and was calculated as the ‘Maximum decrease’ divided by the number of days the minimum was reached. In addition, the final value (_FI) corresponded to the last recorded value before trypanocide treatment. For TWBC, pre-infection and the median value were similarly calculated. The maximum value (TWBC_Max) was the absolute highest level recorded during the course of infection; the maximum increase (TWBC_MI) was the absolute increase and was calculated as the maximum value minus the pre-infection value. The slope increase (TWBC_SI) was the linear trend in TWBC increase and was calculated as the maximum increase divided by the number of days the maximum was reached. In addition, the final value (TWBC_FI) corresponded to the last recorded value before trypanocide treatment. As regards parasitaemia, the average parasitaemia value (PA_Mean) was calculated as the average of the log_10_(parasitaemia + 1) during the course of infection. The maximum parasitaemia (PA_Max) was also noted, as well as the first day post infection (PA_First) that an animal was detected as being parasitaemic for the first time. Finally, the proportion of times an animal was detected as parasitaemic was also recorded (PA_Obs).

**Table 1:** Meaning and results on synthetic phenotypic variables.

					Breed				Test		
Trait	Variable	Variable code	LAG	NDA	BAO	BOR	ZFU	ks_test		Wilcox	
PCV	Pre-infection median	PCV_Init	35.86	34.38	33.80	32.50	37.38	0.083		0.793	
(%)			2.79	0.74	3.63	3.16	3.07				
	Median	PCV_Med	30.36	28.63	27.60	26.13	25.50	0.002	*	0.001	**
			1.25	1.41	2.07	2.85	2.27				
	Minimum	PCV_Min	24.86	23.88	22.60	21.75	19.38	0.004	*	0.001	**
			1.46	1.73	2.30	3.28	2.45				
	Maximum decrease	PCV_MD	-11.00	-10.50	-11.20	-10.75	-18.00	0.010	*	0.003	*
			2.31	2.00	4.32	2.60	4.63				
	Slope decrease	PCV_SD	-0.13	-0.21	-0.18	-0.19	-0.32	0.003	*	0.001	**
			0.02	0.07	0.07	0.07	0.11				
	Final value	PCV_FI	31.86	31.00	30.20	29.13	24.88	0.048		0.007	*
			2.67	2.07	3.27	3.23	5.99				
	Final—init	PCV_FI-Init	-4.00	-3.38	-3.60	-3.38	-12.50	0.033		0.016	*
			4.55	2.07	2.70	3.07	7.48				
PA	Mean [log_10_(PA+1)]	PA_Mean	1.88	2.01	2.17	1.67	2.16	0.425		0.060	
(/ml)			0.22	0.46	0.48	0.94	0.88				
	Rate of positive observation	PA_Obs	0.51	0.53	0.60	0.44	0.55	0.342		0.059	
			0.06	0.11	0.11	0.24	0.21				
	Frist day of positive sample	PA_First	16.00	15.75	15.60	15.38	17.13	0.744		0.362	
			2.08	3.96	3.05	2.50	2.64				
	Maximum value [log_10_(PA+1)]	PA_Max	5.09	5.14	4.96	4.73	5.26	0.750		0.202	
			0.48	0.58	0.53	0.92	0.83				
Hb	Pre-infection median	Hb_Init	12.45	11.51	11.47	11.11	12.78	0.021		0.757	
(g/dl)			0.94	0.36	0.94	0.93	1.16				
	Median	Hb_Med	10.05	9.37	9.08	8.51	8.33	0.001	**	0.001	**
			0.48	0.43	0.47	0.83	0.80				
	Minimum	Hb_Min	8.03	7.61	6.88	7.03	6.33	0.035		0.004	*
			0.92	0.85	1.03	1.33	1.08				
	Maximum decrease	Hb_MD	-4.42	-3.89	-4.59	-4.08	-6.45	0.002	*	0.002	*
			1.07	0.96	0.67	1.08	0.95				
	Slope decrease	Hb_SD	-0.08	-0.06	-0.07	-0.10	-0.11	0.038		0.060	
			0.03	0.02	0.03	0.06	0.03				
	Final value	Hb_FI	10.31	9.85	9.64	9.49	7.75	0.021		0.005	*
			0.51	0.62	1.00	0.96	1.79				
	Final—init	Hb_FI-Init	-2.14	-1.66	-1.83	-1.63	-5.03	0.001	**	0.002	*
			0.77	0.49	0.67	0.78	1.86				
RBC	Pre-infection median	RBC_Init	8.56	8.17	8.28	7.67	9.30	0.004	*	0.964	
(x10^9^/ml)			0.83	0.37	0.58	0.82	0.61				
	Median	RBC_Med	7.16	6.81	6.69	5.82	6.36	0.010	*	0.020	
			0.55	0.79	0.38	0.73	0.60				
	Minimum	RBC_Min	5.82	5.37	4.98	4.53	4.38	0.043		0.014	
			0.76	0.87	0.50	0.99	1.20				
	Maximum decrease	RBC_MD	-2.74	-2.80	-3.30	-3.13	-4.93	0.002	*	0.000	**
			0.92	0.77	0.69	0.77	1.00				
	Slope decrease	RBC_SD	-0.05	-0.05	-0.04	-0.05	-0.09	0.046		0.014	
			0.02	0.02	0.02	0.01	0.04				
	Final value	RBC_FI	8.01	7.34	7.30	6.40	5.44	0.018		0.007	*
			1.17	1.25	0.52	0.97	1.53				
	Final—init	RBC_FI-Init	-0.55	-0.83	-0.98	-1.26	-3.86	0.001	**	0.000	**
			1.41	1.07	0.81	0.87	1.48				
TWBC	Pre-infection median	TWBC_Init	6.43	6.26	7.42	6.70	6.70	0.167		0.699	
(x10^6^/ml)			0.88	0.39	0.93	0.79	0.91				
	Median	TWBC_Med	13.81	12.31	11.70	9.96	8.99	0.001	**	0.000	**
			2.00	2.23	1.33	2.06	1.64				
	Maximum	TWBC_Max	18.27	17.80	16.82	15.09	13.23	0.009	*	0.003	*
			2.78	2.99	2.42	2.45	2.32				
	Maximum increase	TWBC_MI	11.84	11.54	9.40	8.39	6.53	0.010	*	0.002	*
			2.03	3.11	3.12	2.84	2.83				
	Slope increase	TWBC_SI	0.14	0.12	0.09	0.11	0.09	0.143		0.036	
			0.06	0.03	0.02	0.06	0.04				
	Final value	TWBC_FI	16.96	15.21	15.02	13.26	10.63	0.020		0.000	**
			3.02	3.71	2.51	2.97	3.34				
	Final—init	TWBC_FI-Init	10.53	8.95	7.60	6.56	3.93	0.025		0.000	**
			2.27	3.76	3.27	3.22	3.67				

For each breed, the average for the individuals is noted, with its standard deviation below. *P*-values for the non-parametric Kruskal-Wallis tests (ks_test) on the 5 breeds and for the Wilcoxon tests (wilcox) comparing LAG and ZFU (LAG>ZFU hypothesis except PA) are shown.

Symbols:

“*” *P*-values ≤ 0.01

“**”*P*-values ≤ 0.001

The breed effect was assessed for these synthetic variables using the non-parametric Kruskal-Wallis test (for a comparison of all breeds) and the one-sided hypothesis Wilcoxon test for the LAG-ZFU comparison, and a comparison of variances was performed using the non-parametric Fligner-Killeen test. A Spearman rank correlation test was performed between a subset of 20 variables, four for each five traits, PCV, PA, Hb, RBC and TWBC (displayed in Table 2) to avoid redundant variables. A principal components analysis (PCA) was carried out on the same 20 variables using the Ade4 R package [42].

**Table 2:** Results of the linear mixed effects model on PCV.

Fixed effects	Estimate	S.E.	p-value	
Intercept (LAG)	33.30	1.14	0.00000	***
DPI (LAG)	-0.04	0.05	0.43270	
DPI^2^ (LAG)	-0.0010	0.0007	0.12770	
DPI^3^ (LAG)	0.00001	0.00000	0.00100	**
BAO	-0.64	1.76	0.71930	
BOR	-2.85	1.54	0.07360	
NDA	0.92	1.54	0.55370	
ZFU	1.37	1.57	0.38960	
DPI:BreedBAO	-0.12	0.08	0.14310	
DPI:BreedBOR	-0.15	0.07	0.03360	*
DPI:BreedNDA	-0.17	0.07	0.01600	*
DPI:BreedZFU	-0.35	0.07	0.00000	***
DPI^2^:BreedBAO	0.0019	0.0010	0.05920	
DPI^2^:BreedBOR	0.0028	0.0008	0.00090	***
DPI^2^:BreedNDA	0.0026	0.0008	0.00230	**
DPI^2^:BreedZFU	0.0053	0.0009	0.00000	***
DPI^3^:BreedBAO	-0.00001	0.00000	0.05490	
DPI^3^:BreedBOR	-0.00001	0.00000	0.00030	***
DPI^3^:BreedNDA	-0.00001	0.00000	0.00340	**
DPI^3^:BreedZFU	-0.00002	0.00000	0.00000	***

For the different fixed effects, estimates, standard errors (S.E.) and associated *P*-values are indicated. LAG was the reference breed so the intercept and DPI values are estimated for this breed.

Symbols:

“*” *P*-values ≤ 0.05

“**”*P*-values ≤ 0.01

“***”*P*-values ≤ 0.001

### Statistical analyses on longitudinal data

Then, to benefit from the repeated measure taken on each head of cattle for 5 months we performed mixed linear models that accounted for intra-correlation measurements for each animal and assessed the breed effect. PCV and TWBC could be modelled as Gaussian variables using the NLME R package [51].

Firstly, PCV was the response variable, the animal was considered as a random effect, and the breed and days post infection (DPI) were fixed effects, possibly with interactions. Following the recommendations made in [52], several models were tested using a Maximum Likelihood (ML) algorithm and the one which seemed to best fit the data was computed using REML (Restricted Maximum Likelihood). Random effects were compared using likelihood ratio tests and fixed effects were chosen according to an individual *t*-test and single model Anova (F-test). Diagnostic plots were edited to check model assumptions.

The final model for PCV was:

$$PCV_{ijk}=\mu +B_{i}\times \left(t_{j}+t_{j}^{2}+t_{j}^{3}\right)+a_{k}\times \left(t_{j}+t_{j}^{2}\right)+\varepsilon_{ijk}$$ (1)

where *PCV*_*ijk*_ was the PCV of an animal *k* from breed *i* on *t*_*j*_, with *a*_*k*_ representing the animal (*k* = 1, …, 36), *B* (*j =* 1, …, 5) the breed, and *t* the time after infection as numeric (63 dates in total, from 0 to 133 DPI). The PCV values at *t* = 0 were calculated as the median of the five pre-infection values. Within-animal errors *ε*_*ijk*_ followed a Gaussian distribution *N*(0, σ^2^ *δ* ^2^_*Bi*_), allowing the modelling of heteroscedasticity among breeds (*δ* ^2^_*Bi*_), and autocorrelation within animal errors (cor (*ε*_*kj*_, *ε*_*kj’*_)) was modelled with an autoregressive model AR(1). Random effects were also normally distributed and *a*_*k*_ ~ *N*(0,ψ), where ψ was the symmetric variance-covariance matrix of size (3 x 3), with variances of the three random effects on the diagonal and their co-variances outside the diagonal.

After graphical observations, TWBC was modelled according to an asymptotic regression model computed in the *nlme* R package [51], which followed the standard equation:

$$y\left(x\right)=\phi_{1}+\left(\phi_{2}-\phi_{1}\right)\times exp\left[-exp\left(\phi_{3}\right)x\right]$$ (2)

so that *Ф*_*1*_ is the asymptote as *x* → ∞, *Ф*_*2*_ is *y*(0) and *Ф*_*3*_ is the logarithm of the rate constant. Applied to TWBC, this equation became:

$$WC_{ijk}=\left(\phi_{1}+B_{1i}+a_{1k}\right)+\left[\left(\phi_{2}+B_{2i}\right)-\left(\phi_{1}+B_{1i}+a_{1k}\right)\right]\times exp\left[-exp\left(\phi_{3}+a_{3k}\right)t_{j}\right]+\varepsilon_{ijk}$$ (3)

where *WC*_*ijk*_ was the TWBC of an animal *k* from breed *i* on *t*_*j*_, *B*_1*i*_ and *B*_2*i*_ were the breed fixed effects, and *a*_1*k*_ and *a*_2*k*_ were the animal random effects. Within-animal errors *ε*_*ijk*_ followed a Gaussian distribution *N*(0, σ^2^ *δ* ^2^_*Bi*_), allowing modelling of the heteroscedasticity among breeds, and autocorrelation within animal errors (cor(*ε*_*kj*_, *ε*_*kj’*_)) was modelled with an autoregressive model AR(1); and *a*_*k*_ ~ *N*(0,ψ), where ψ was the symmetric variance-covariance matrix of size (2 x 2), with variances of the two random effects on the diagonal and their co-variances outside the diagonal.

Parasitaemia dynamics can be investigated working either with a Bernouilli variable (0 = non-parasitaemic; 1 = parasitaemic) or with a quantitative variable to try inferring the level of parasitaemia. We used the glmmADMB R package [53], which offers flexible ways of running mixed models for non-Gaussian variables. Firstly, we modelled parasitaemia as a simple Bernouilli variable, using a binomial distribution with a logit link: $logit\left(\mu_{ijk}\right)=log\left(\frac{Pr\left(Y_{ijk}=1\right)}{Pr\left(Y_{ijk}=0\right)}\right)$

$$logit\left(\mu_{ijk}\right)=\mu +B_{i}\times t_{j}+t_{j}^{2}+t_{j}^{3}+t_{j}^{4}+t_{j}^{5}+a_{k}\times t_{j}+\varepsilon_{ijk}$$ (4)

where *μ*_*ijk*_ was the probability of a head of cattle *a*_*k*_ being positive for parasitaemia, *B*_*i*_ the breed effect, *t*_*j*_ the time, *ε*_*ijk*_ the residuals, and *a*_*k*_ the random animal effect.

Secondly, the parasitaemia level was investigated as count data provided by buffy coat observations. This variable displayed strong over-dispersion. Thus a negative binomial, instead of a Poisson distribution, was used to cope with over-dispersion with *Var*(*X*) = *E*(*X*) × (1 + *E*(*X*) / *α*). The model was:

$$PA_{ijk}=\mu +t_{j}+t_{j}^{2}+t_{j}^{3}+t_{j}^{4}+t_{j}^{5}+a_{k}\times t_{j}+\varepsilon_{ijk}$$ (5)

where *PA*_*ijk*_ was the count of parasites for a head of cattle *a*_*k*_, *t*_*j*_ the time, *ε*_*ijk*_ the residuals, and *a*_*k*_ the random animal effect.

## Results

### Genetic assignment of the experimental cattle to local breeds

The individual-based PCA (S1 Fig) revealed that newly sampled individuals (namely NDAa, BAOa, LAGa, BORa and ZFUa populations in the supplementary data) were located as expected near individuals of their reference breed previously genetically characterized, except for two BAOa individuals which were close to the BOR breed. These results were confirmed by the unsupervised hierarchical clustering analysis with k = 3 clusters (S1 Fig and S1 Table). Only the two BAOa individuals identified by PCA presented a higher level of ZEB ancestry (0.19 and 0.28, respectively) than individuals from the BAO breed (0.01 on average). These animals, erroneously assigned to BAO according to their phenotypic characteristics, were discarded from the further analyses.

### Infection development

Phenotypic data from 36 cattle, five BAO, eight BOR, seven LAG, eight NDA and eight ZFU, were then studied. In fact, one Baoulé displaying trypanosomes in the blood on the day of its arrival at CIRDES before the experiment was treated and removed from the phenotypic analyses. The raw phenotypic data are presented in S2 Table.

**Fig 2:**
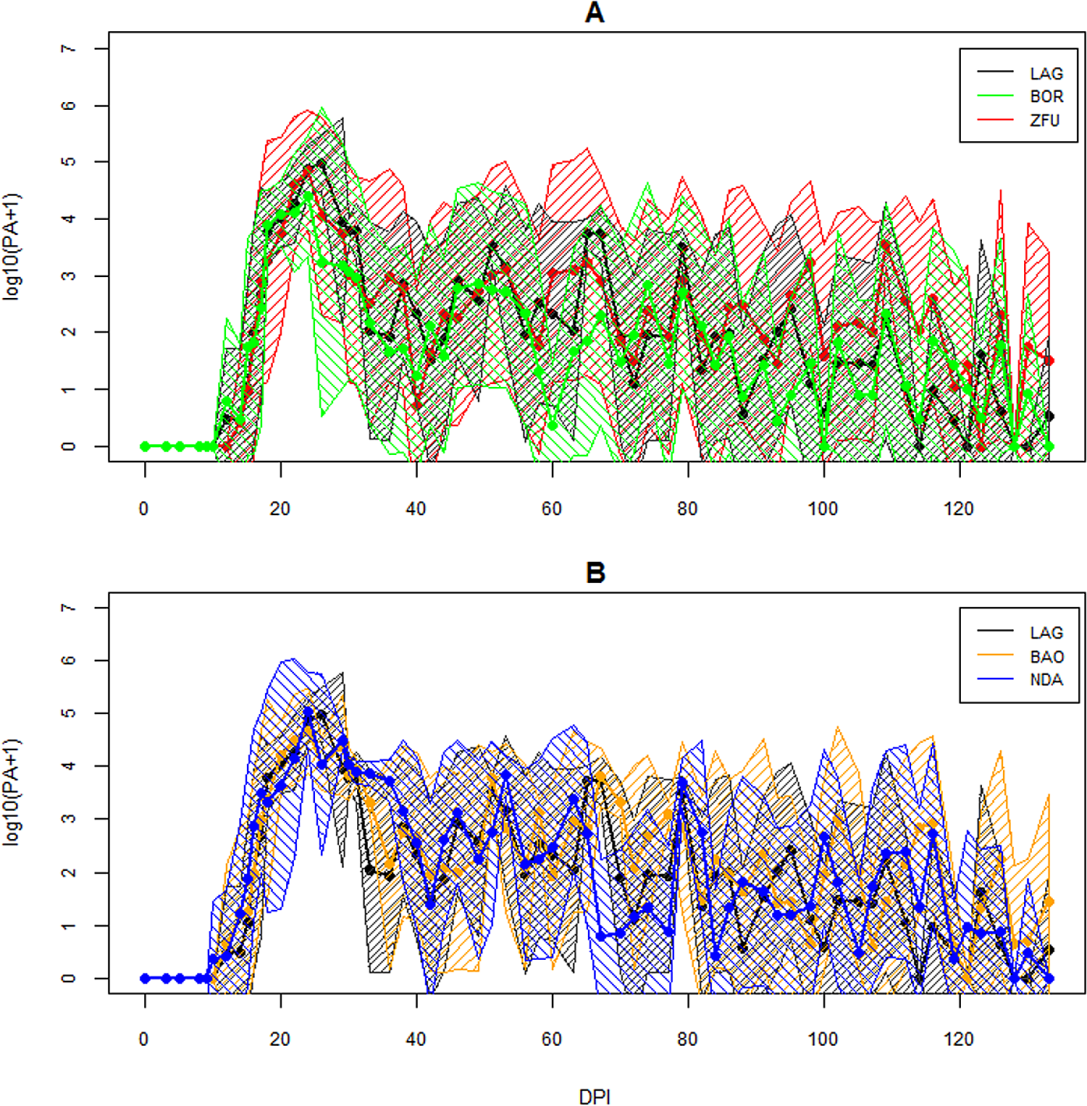
Evolution of parasitaemia over the time course of infection. For visual convenience, the five breeds are presented in two graphical devices, with LAG used as the reference population. PA is estimated as the number of trypanosomes/ml of blood. A. log_10_(PA+1) evolution for the LAG, ZFU and BOR breeds. B. log_10_(PA+1) evolution for the LAG, NDA and BAO breeds. The continuous lines are the average per breed and the surrounding areas represent more and less one standard deviation from the mean.

**Fig 3:**
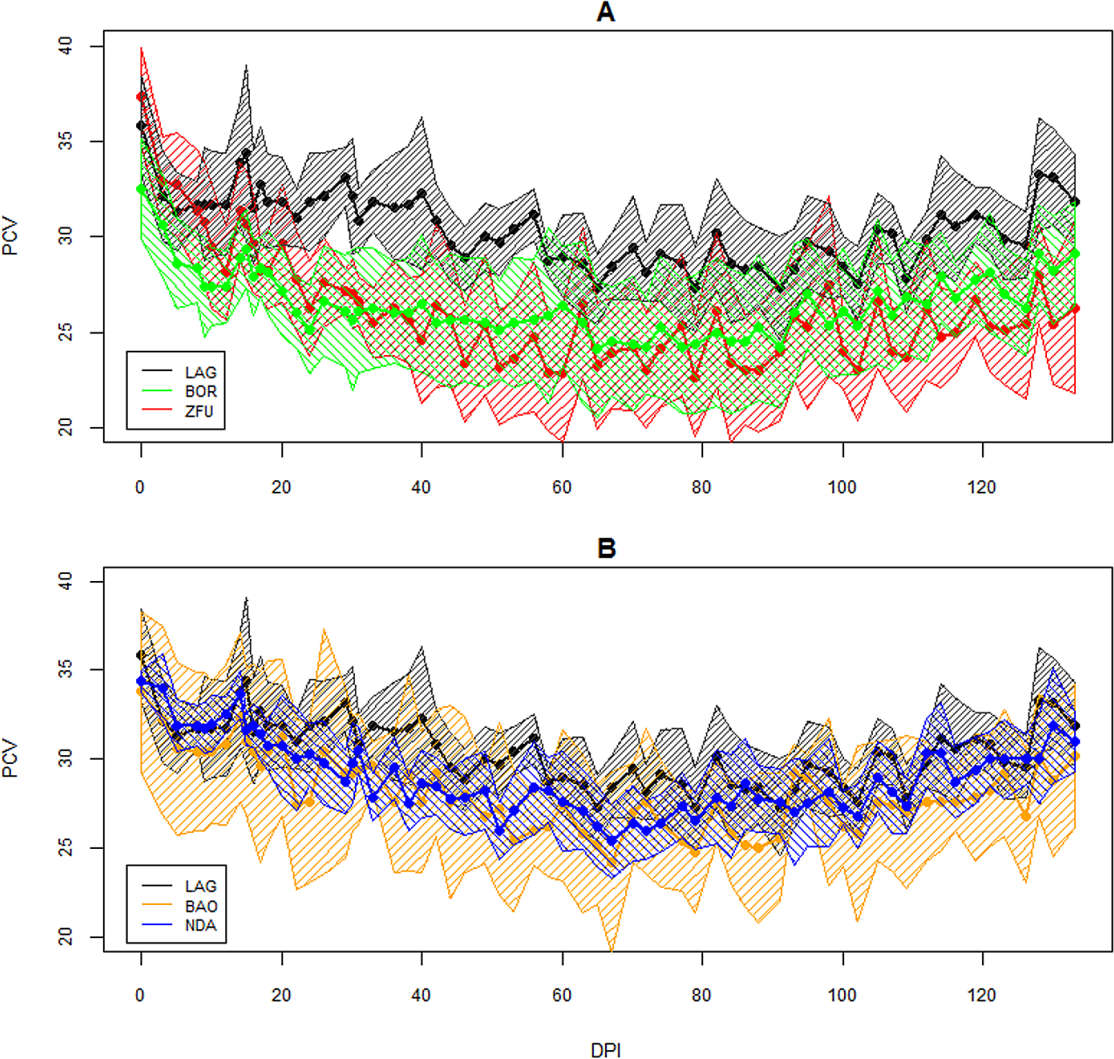
Evolution of PCV over the time course of infection. PCV is expressed as a percentage of blood volume. A. PCV evolution for the LAG, ZFU and BOR breeds. B. PCV evolution for the LAG, NDA and BAO breeds. The continuous lines are the average per breed and the surrounding areas represent the confidence intervals of the means at 95%.

**Fig 4:**
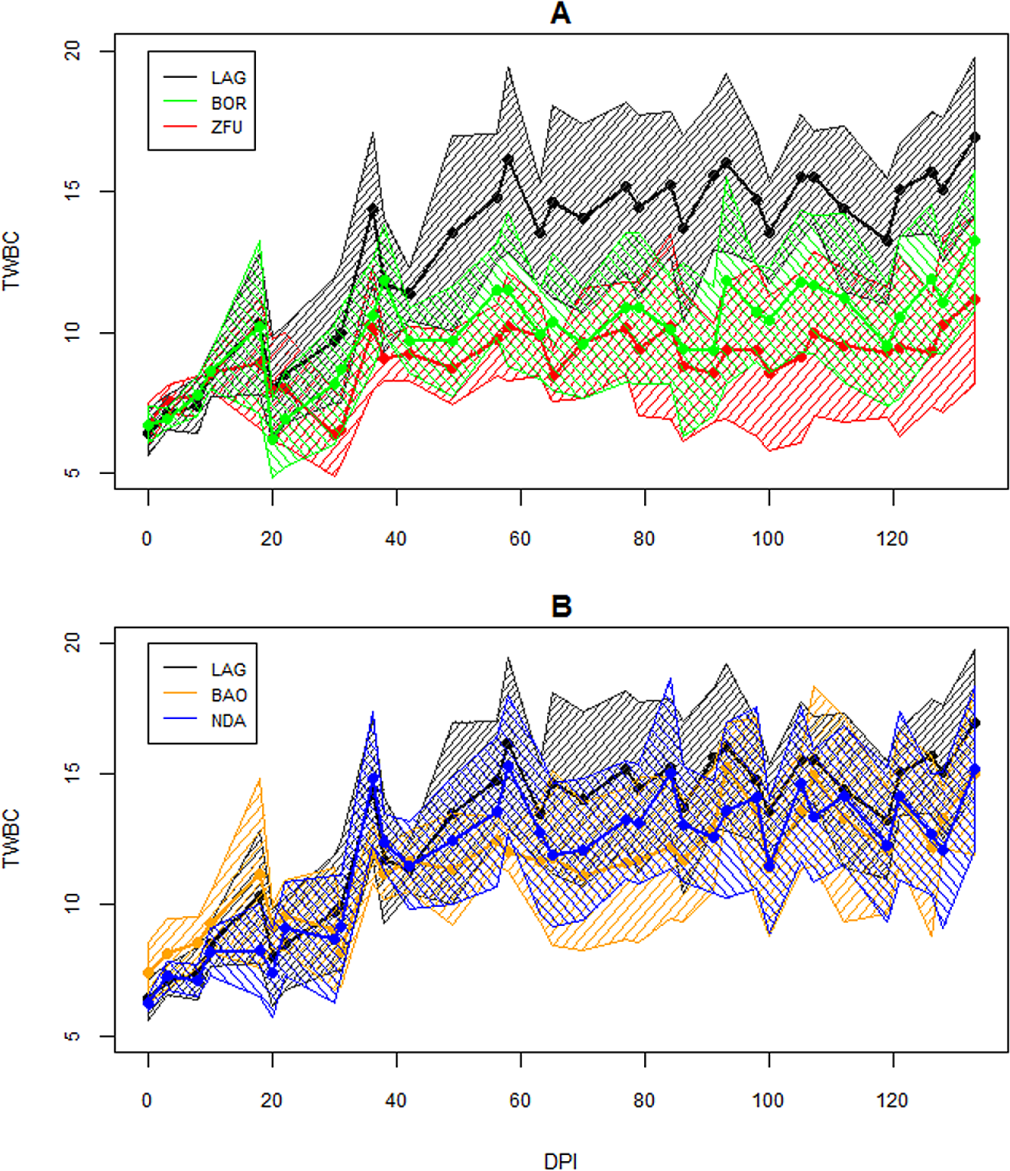
Evolution of TWBC over the time course of infection. TWBC is expressed as million leukocytes/ml of blood. A. TWBC evolution for the LAG, ZFU and BOR breeds. B. TWBC evolution for the LAG, NDA and BAO breeds. The continuous lines are the average per breed and the surrounding areas represent the confidence intervals of the means at 95%.

**Fig 5:**
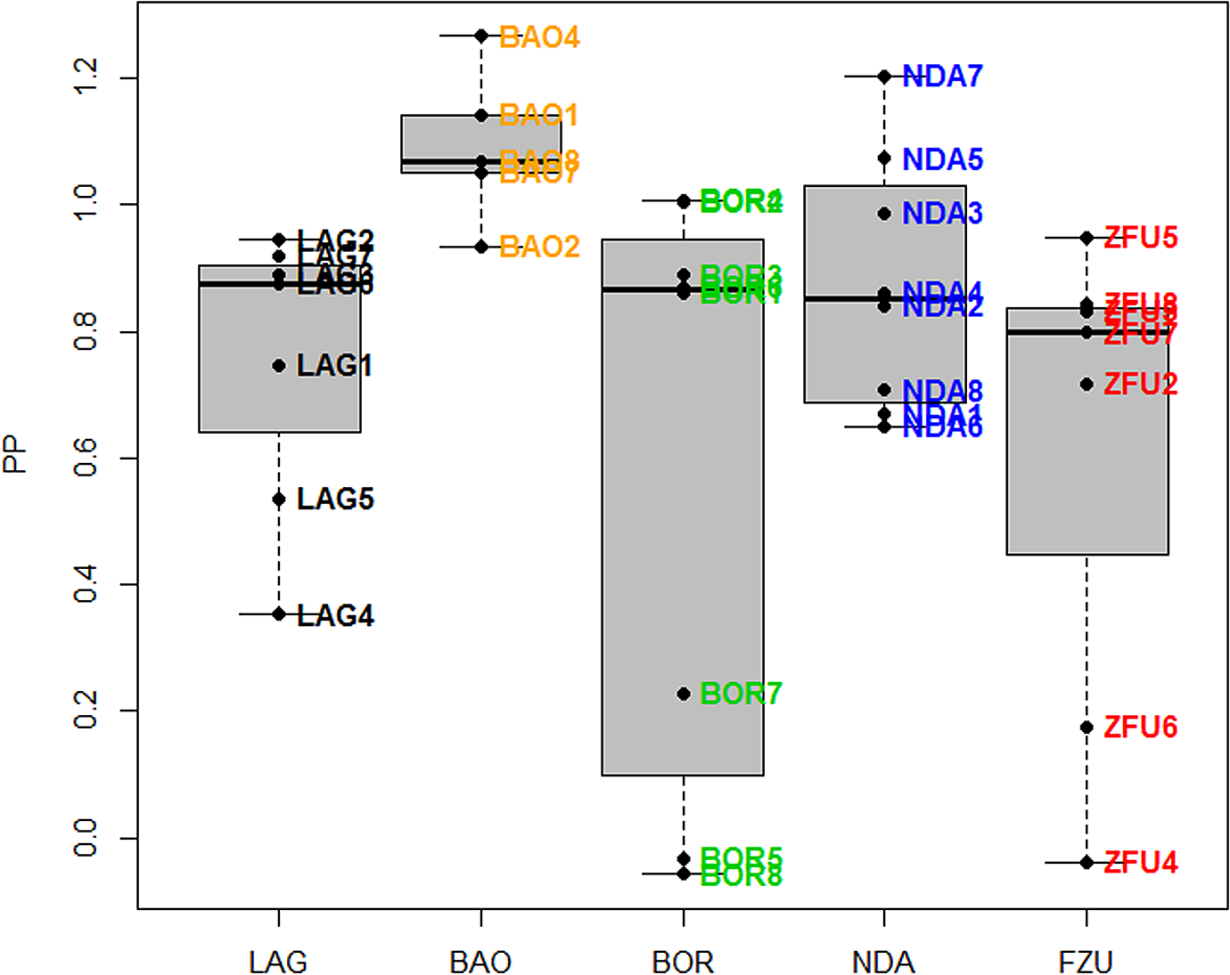
PP on IgG directed against trypanosome antigens at the end of the experiment depending on the breed. Boxplot representing PP for the five breeds at DPI = 133, with the cattle codes.

As the PCV of one ZFU dropped to 15% at 95 DPI, that individual was treated with aceturate diminazene and this date was considered as its last observation. The infection followed a classic course, as expected for inoculation with *T*. *congolense* IL1180. The changes in parasitaemia, PCV and TWBC are displayed in Figs 2–4. Parasites were detected in the blood between 10 and 24 DPI. Generally, peaks were reached between 20 and 30 DPI and then parasitaemia waves were observed. PCV dropped more or less depending on the cattle and minima were reached between 26 and 109 DPI, and increased slowly after that, except for two ZFU whose PCV decreased until treatment. Hb and RBC followed the same dynamics as PCV (data not shown). TWBC increased globally, but a strong variation between breeds was observed. PP increased around 20 DPI, except for two BOR (BOR5 and BOR8) and two ZFU (ZFU4 and ZFU7), whose values remained low during the course of infection (see Fig 5). No reticulocyte was detected during the experiment in any animals.

### The results on synthetic variables highlighted differences in anaemia-related traits and TWBC

Synthetic variables, related to PCV, Hb, RBC, TWBC and PA, were used to assess inter-breed variability and study correlations (see Table 1). PP was not studied as a quantitative variable, since the linearity of PP responses in relation to antibody concentration has not been validated with the method proposed by [50] and [34].

The means and standard deviations per breed for 32 indicators are presented in Table 1, with the *P*-values of the Kruskal-Wallis and Wilcoxon tests. Pre-infection values for PCV, Hb and TWBC were not significantly different between breeds; only initial values for RBC were different (*P*-value = 0.005), with a larger value for ZFU.

The anaemia indicators (PCV_Med, PCV_Min, PCV_MD, PCV_SD, Hb_Med, Hb_MD, Hb_FI-Init, RBC_Med, RBC_MD, RBC_FI-Init) were significantly different between breeds, with usually the best values for LAG, followed by NDA, BAO, BOR and the worst ones for ZFU. In addition, when comparing only LAG and ZFU, the final values for PCV, Hb and RBC before treatment were significantly higher in LAG than in ZFU. The parasitaemia indicators did not significantly differ between breeds. The TWBC indicators were significantly different (TWBC_Med, _Max, _MI) and were highest for LAG, followed by NDA, BAO, BOR, and lastly by ZFU.

**Fig 6:**
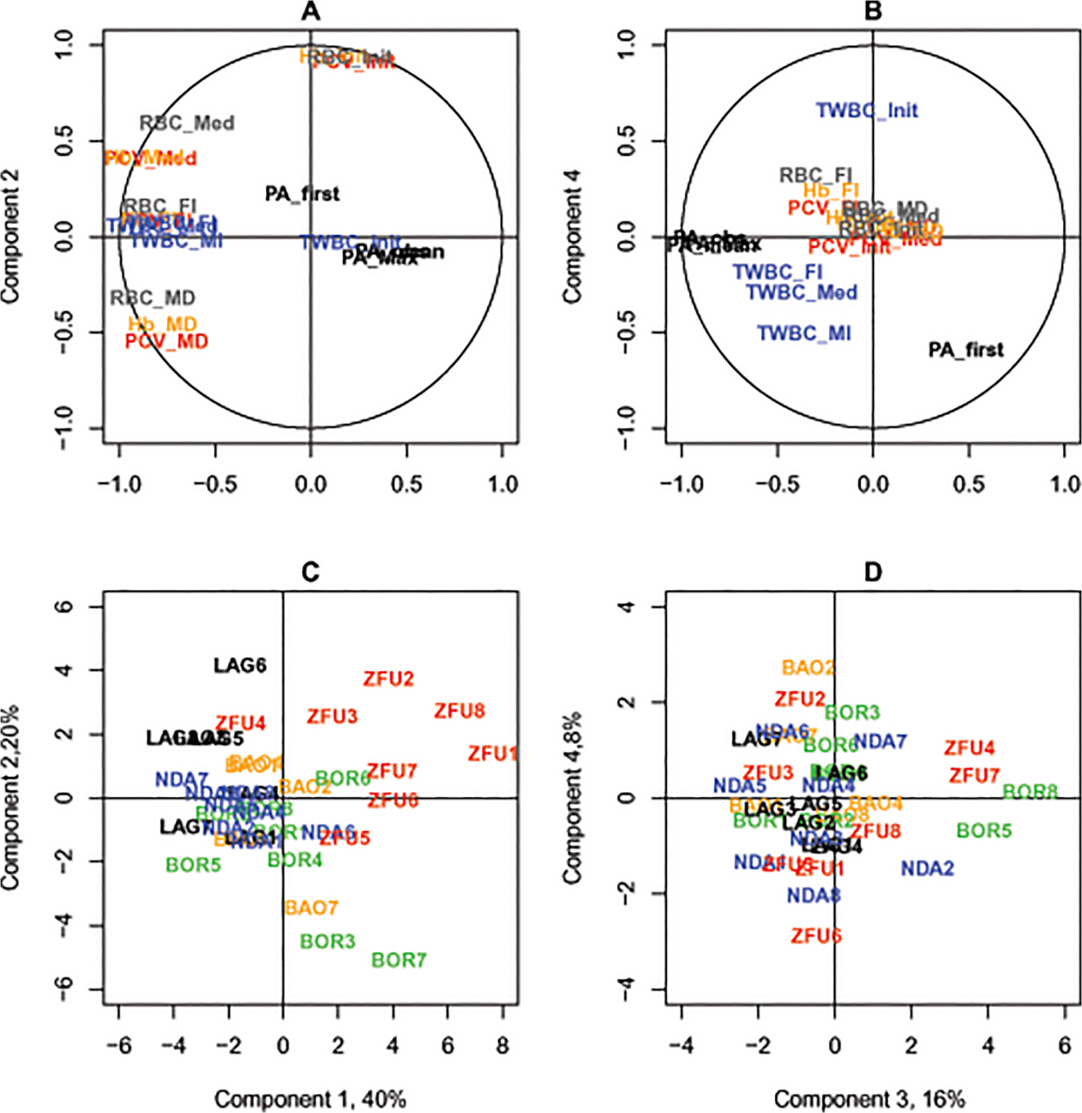
PCA analyses of synthetic variables. A. Correlation circle of the variables used for the PCA, projected on the first two components. B. Correlation circle of the variables used for the PCA, projected on the third and fourth components. PCV-related traits are presented in red, RBC in grey, Hb in orange, TWBC in blue and PA in black. The codes are explained in Table 1. C. Projection of individual coordinates on axes 1 and 2. D. Projection of individual coordinates on axes 3 and 4. Breed colours for LAG, BAO, NDA, BOR and ZFU are respectively black, orange, blue, green and red.

The PCA results are displayed in Fig 6. Axis 1 of the PCA represents anaemia indicators during infection, axis 2 PCV, Hb and RBC pre-infection values and axis 3 parasitaemia (average, maximum value and percentage of positive values). The correlation circles showed information identical to the correlation coefficients estimated between the variables and their associated *P*-values, which are shown in S3 Table. The anaemia indicators, based on PCV, RBC and Hb, were highly correlated, especially the median, maximum decrease and last values between them. They were not correlated to the pre-infection variables, except for the maximum decrease indicators (MDs), which were linked to the initial values by construction. The parasitaemia indicators (average level and percentage of positive values) were significantly correlated to the anaemia indicators and, in particular, negatively correlated with median PCV during infection (*ρ* = -0.56, *P* = 4.10^–4^). The TWBC indicators were correlated with each other during infection (and not with pre-infection values) and were positively associated with PCV, Hb and RBC (i.e. median TWBC and median PCV: *ρ* = 0.59, *P* = 10^–4^) but they were not correlated to parasitaemia.

When positioning individuals on the factorial planes, we found that the ZFU animals were mainly on the right-hand side of Axis 1, highlighting their greatest anaemia; LAG and NDA were found on the left-hand side of the axis (least anaemia), and BAO and BOR between them, with a high dispersion for BOR. Two BOR individuals (BOR3 and BOR7) drew axis 2 with lower PCV, Hb and RBC pre-infection values. On Axis 3, no trend by breed was found, but two ZFU, two BOR and one NDA drew this axis. These animals displayed a lower average parasitaemia and percentage of positive samples, especially the two BOR (BOR5 and BOR8), which displayed only 4 and 5 dates with detectable parasites during the experiment. Such intra-breed variability has been reported in different breeds, BAO and ZFU [25], [26], [54], in East African zebu [55] and in N’Dama [56].

### Longitudinal analyses of PCV, TWBC and parasitaemia

PCV was modelled during the course of infection according to a degree 3 polynomial in order to capture its decrease and its recovery [19].The model (1) result can be found in Table 2. LAG was used as the reference breed as a contrast. LAG seemed mildly affected by the infection, since coefficients on DPI and DPI^2^ were negative but not significantly different from zero; the coefficient on DPI^3^ was slightly positive and significant. For the other breeds, the coefficients on the intercept were not significantly different from zero, meaning there was no significant breed difference on pre-infection PCV. On the other hand, numerous interactions between DPI and the breed were significant, especially the strongly negative interaction between DPI*ZFU, highlighting the greater drop in PCV in ZFU in comparison to LAG, with an estimate I(DPI*ZFU) = -0.35. Negative and slightly significant interactions, DPI*NDA and DPI*BOR, were also noticed. To allow recovery, positive interactions between DPI^2^ existed for these breeds, compensated for by a negative interaction with DPI^3^. The adjusted BAO estimates were not significantly different from LAG.

For almost all the cattle, TWBC increased immediately after infection and capped at a maximum value, and it was thus modelled by an asymptotic function (model 3) whose results are in Table 3. The initial value estimate was 5.7*10^6^ TWBC/ml (S.E. = 0.5) for LAG, its asymptotic estimate was 15.8*10^6^ TWBC/ml (S.E. = 0.9) and its logarithm rate constant was -3.6 (S.E. = 0.1). The initial values for BAO and ZFU were slightly and significantly larger than for LAG. Interestingly, the asymptotic estimates for BOR and ZFU were significantly lower than for LAG, with a decrease of -4.4 (S.E. = 1.2, *P* = 3.10^–4^) and -6.2 (S.E. = 1.2, *P*<10^–4^) for these breeds, respectively, in comparison with LAG. The NDA and BAO asymptotic estimates were not different from the LAG estimate.

**Table 3:** Results of the non-linear mixed effects model on TWBC.

Fixed effects	Estimate	S.E.	*P*-value	
*Ф*_*1*_ (LAG)	15.8460	0.9197	0.0000	***
*Ф*_*1*_.BreedBAO	-2.6275	1.3490	0.0517	
*Ф*_*1*:_BreedBOR	-4.3733	1.2117	0.0003	***
*Ф*_*1*:_BreedNDA	-1.5012	1.1938	0.2088	
*Ф*_*1*:_BreedZFU	-6.2201	1.2304	0.0000	***
*Ф*_*2*_.(LAG)	5.6983	0.5167	0.0000	***
*Ф*_*2 *_:BreedBAO	1.6068	0.6711	0.0168	*
*Ф*_*2 *_:BreedBOR	0.8543	0.6477	0.1875	
*Ф*_*2*_:BreedNDA	0.2475	0.6813	0.7165	
*Ф*_*2 *_:BreedZFU	1.4240	0.6219	0.0222	*
*Ф*_*3*_	-3.6259	0.1103	0.0000	***

Estimates, standard errors (S.E.) and associated *P*-values are indicated for the non-linear coefficients *Ф*_*1*_ (asymptote), *Ф*_*2*_ (starting value) and *Ф*_*3*_ (rate constant) and interactions with breeds.

Symbols:

“*” *P*-values ≤ 0.05

“**”*P*-values ≤ 0.01

“***”*P*-values ≤ 0.001

The probability of being positive for parasitaemia was investigated according to model (4) and the result is shown in Table 4. The different degrees of the time polynomial were highly significant, to fit the wave form of the probability of positive parasitaemia. Interestingly, a positive and significant interaction was found between DPI and the ZFU breed (estimate = 0.0196, *P* = 0.002), showing a trend of ZFU cattle to remain positive for parasitaemia longer than LAG cattle. For instance, the odds ratio of ZFU/LAG was estimated at 2.1 at 100 DPI. The other breeds did not show any parameters significantly different from LAG. No breed effect was highlighted on the parasite count (model 5).

**Table 4:** Results of the generalized linear mixed effects model on parasitaemia.

Fixed effects	Estimate	S.E.	*P*-value	
Intercept (LAG)	-12.2000	1.1200	2.00E-16	***
DPI (LAG)	1.3600	0.1180	2.00E-16	***
BAO	0.0161	0.6960	0.9815	
BOR	-0.5350	0.6200	0.3881	
NDA	0.0698	0.6150	0.9096	
ZFU	-1.2000	0.6150	0.0512	.
DPI^2^ (LAG)	-0.0448	0.0044	2.00E-16	***
DPI^3^ (LAG)	0.0006	0.0001	2.00E-16	***
DPI^4^ (LAG)	-0.000004	0.0000	3.40E-15	***
DPI^5^ (LAG)	0.00000001	0.0000	2.90E-12	***
DPI:BreedBAO	0.0073	0.0071	0.3075	
DPI:BreedBOR	-0.0011	0.0067	0.8708	
DPI:BreedNDA	0.0006	0.0064	0.9268	
DPI:BreedZFU	0.0196	0.0064	0.0024	**

Estimates, standard errors (S.E.) and associated *P*-values are indicated for the different fixed effects.

Symbols:

“*” *P*-values ≤ 0.05

“**”*P*-values ≤ 0.01

“***”*P*-values ≤ 0.001

## Discussion

This study set out to investigate trypanotolerance traits in five West African local breeds, including three breeds, LAG, BAO and BOR, that had yet to be extensively studied.

Studying PCV, which is considered as a good indicator for simply assessing trypanotolerance as anaemia is the main pathogenic feature of infection by *T*. *congolense* and is correlated to growth [23], [19], [57], and to reproductive performances [58], we first confirmed the trypanosusceptibility status of ZFU and the trypanotolerant status of NDA.

Interestingly, under our experimental conditions, our experiment also showed that shorthorn taurines, the LAG and BAO breeds, are as trypanotolerant as the longhorn NDA. Indeed, shorthorn taurines controlled PCV remarkably well, as exemplified by LAG, which displayed the best anaemia control indicators. Depending on breed history and agroecology, the strong trypanotolerance of LAG is not surprising, since this breed inhabits a region under a Sudano-Guinean climate infested by several tsetse species from the *morsitans*, *palpalis* and *fusca* groups [59], [60], and has thus been under strong selective pressure for hundreds of generations. BOR, as expected according to its admixed origin between ZFU and shorthorn taurine and its breeding conditions in a sub-humid area [30], displayed intermediate trypanotolerance between AFT and AFZ. To qualify in our comparisons between LAG and NDA, the infected NDA cattle originated from Madina-Diassa in Mali, and not from Fouta-Djallon in Guinea, which is assumed to be the cradle of the breed [12]. Genetic differentiation was reported between NDA from Guinea and Mali, with the latter having some alleles of indicine origin [40]. Thus NDA from Guinea might be more trypanotolerant than NDA from Mali, and would deserve to be better characterized at phenotypic level.

No reticulocytes were detected in our experiment, whereas they were observed by [61] with large variations between breeds. Reticulocytes, which are normally not present in ruminant blood, appeared late during anaemia and their absence prevented any conclusion as to the status of the anaemia (regenerative or not, [62]). Here, anaemia was not very pronounced and maybe not sufficient to induce a detectable occurrence of reticulocytes in blood.

The parasitaemia results were less conclusive than those for PCV. No comparison using synthetic variables was significant, but according to the linear mixed model based on the longitudinal data, ZFU harboured detectable parasites longer than LAG, whereas differences in the parasitaemia scores were not found. Our results correspond to those described in [19], which did not highlight differences in either the prepatent period or in the first parasitaemia waves between NDA and the Boran Zebu, but more persistent infection was found in the Boran during primo-infection.

Although most of the parasitaemia indicators were not significantly different between breeds, we found a significant negative phenotypic correlation between median PCV during infection and parasitaemia. Such correlations have already been observed in several studies [19], [63], [23], [21] but with different magnitudes depending on the variable calculation, the cattle populations or the experimental conditions. It is obvious that parasite infection is the primary cause of anaemia, but the intensity of the latter is not a simple function of parasite burdens: it depends on interacting mechanisms, including host-inflammatory-mediated anaemia and parasite-induced anaemia [64], more or less buffered by the erythropoietic response [65] or the cell-membrane composition of RBC [66]. Thereby, some QTL were associated with both PCV and the parasitaemia indicators, whereas others were independently linked to one or the other [21]. Moreover, using an experiment with NDA, the Boran Zebu and twins sharing haematopoietic chimaerism, [22] concluded that parasitaemia control and PCV maintenance were due to independent physiological mechanisms, the first one being not linked to the haematopoiesis cells line alone, and the second one depending on hematopoietic stem cells.

Leukocytosis was found in the different breeds, but with significant differences in amplitude between breed types, as ceiling values were maximal for LAG, NDA and BAO, followed by BOR, then ZFU. Lymphocytosis has been reported in NDA but lymphopaenia and neutropaenia were found in Boran cattle by [19] and [22]. Leukopenia is usually thought to be induced by trypanosomoses [67], but here, leukocytosis was found even in susceptible ZFU. These variable features depend on the trypanosome strain [68], the breed and the individual and provide a reminder that infection characteristics are due to complex interactions between host cells and parasites.

Our study also strengthens the hypothesis of a link between the immune response and anaemia control proposed by [22]. Indeed, [22] also found that chimaeras with low PCV had low TWBC counts in comparison with NDA, and TWBC counts were independent from parasitaemia. Our data showed a significant positive correlation between PCV and TWBC, whereas TWBC values were not associated with parasitaemia control. Moreover, [22] suggested that differences in antibody titres between trypanotolerant and trypanosusceptible cattle may be a consequence and not a cause of differences in parasitaemia. Here, using ELISA detecting bovine IgG directed against total trypanosome antigens, it seemed that low parasitaemia resulted in low IgG titres directed against total antigens, which was the case for the two BOR and the ZFU with transient parasitaemia and which did not elicit an increase in PP (BOR5, BOR8 and ZFU4). However, before concluding on the absence of a role of antibodies in controlling parasitaemia, more specific studies on antibodies directed against specific parasite proteins would be needed.

Our study demonstrated the value of shorthorn taurine breeds, and especially Lagune, in relation to its strong adaptation to harsh environments, represented here by trypanosomoses. Specific conservation plans need to be established for these breeds. We need now to determine if similar or different molecular mechanisms and genetic polymorphisms underlie trypanotolerance in shorthorn and longhorn taurines, which have different demographic histories [16]. Deciphering these molecular mechanisms using accurate immunological studies, and genome or transcriptome analyses, could improve knowledge on host-pathogen interactions and their evolution and is a prerequisite for developing new ways of controlling trypanosomoses.

## Supporting Information

S1 Fig PCA and unsupervised hierarchical clustering results. A. PCA results. The individuals are plotted on the first two principal components according to their coordinates. Ellipses characterize the dispersion of each breed around its centre of gravity. The EUT, AFT and ZEB breeds are plotted in red, green and blue, respectively. The EUTxAFT, EUTxZEB and AFTxZEB admixed breeds previously sampled are shown in yellow and the newly sampled populations (BAOa, BORa, LAGa, NDAa and ZFUa) in black. B. Unsupervised hierarchical clustering results for the 545 individuals genotyped for 43,845 SNPs with an inferred number of clusters k = 3 obtained with Admixture 1.04. For each individual, the proportions of each cluster (y axis) which were interpreted as representative of EUT, AFT and ZEB ancestries are plotted in red, green and blue, respectively.
(PDF)

S1 Table Results of the unsupervised hierarchical clustering. For each newly sampled breed and for its corresponding cattle breed that was previously genetically characterized [29], the average of cluster 1, 2 and 3 individual proportions, obtained with Admixture 1.04 (k = 3), are given and interpreted as representative of EUT, AFT and ZEB ancestries respectively.
(XLSX)

S2 Table Raw phenotypic data. Breed; Ani = Animal; DPI = Day Post Infection; PCV = Packed Cell Volume; PA = Parasites in number of trypanosomes/ml; PA_count = Parasites in number of trypanosomes/microscopic observation; EUT = Percent assignment to cluster EUT with 54K SNP chip; AFT = Percent of assignment to cluster AFT with 54K SNP chip; ZEB = Percent of assignment to cluster ZEB with 54K SNP chip; TWBC = White cell rate (millions per ml); RBC = Red blood cell rate (billion per ml); Hb = Haemoglobin rate (g/dl); PP = Percentage of relative Positivity using indirect Elisa.
(XLSX)

S3 Table Correlation between synthetic variables. Correlation values are indicated above the diagonal and *P*-values below the diagonal.
(XLSX)
