# Supplementary figures and images for: A Comparison of Phenotypic Traits Related to Trypanotolerance in Five West African Cattle Breeds Highlights the Value of Shorthorn Taurine Breeds
Source: PLoS One. 2015 May 8;10(5):e0126498. doi: 10.1371/journal.pone.0126498 (PMC4425517; doi:10.1371/journal.pone.0126498)

**A.**

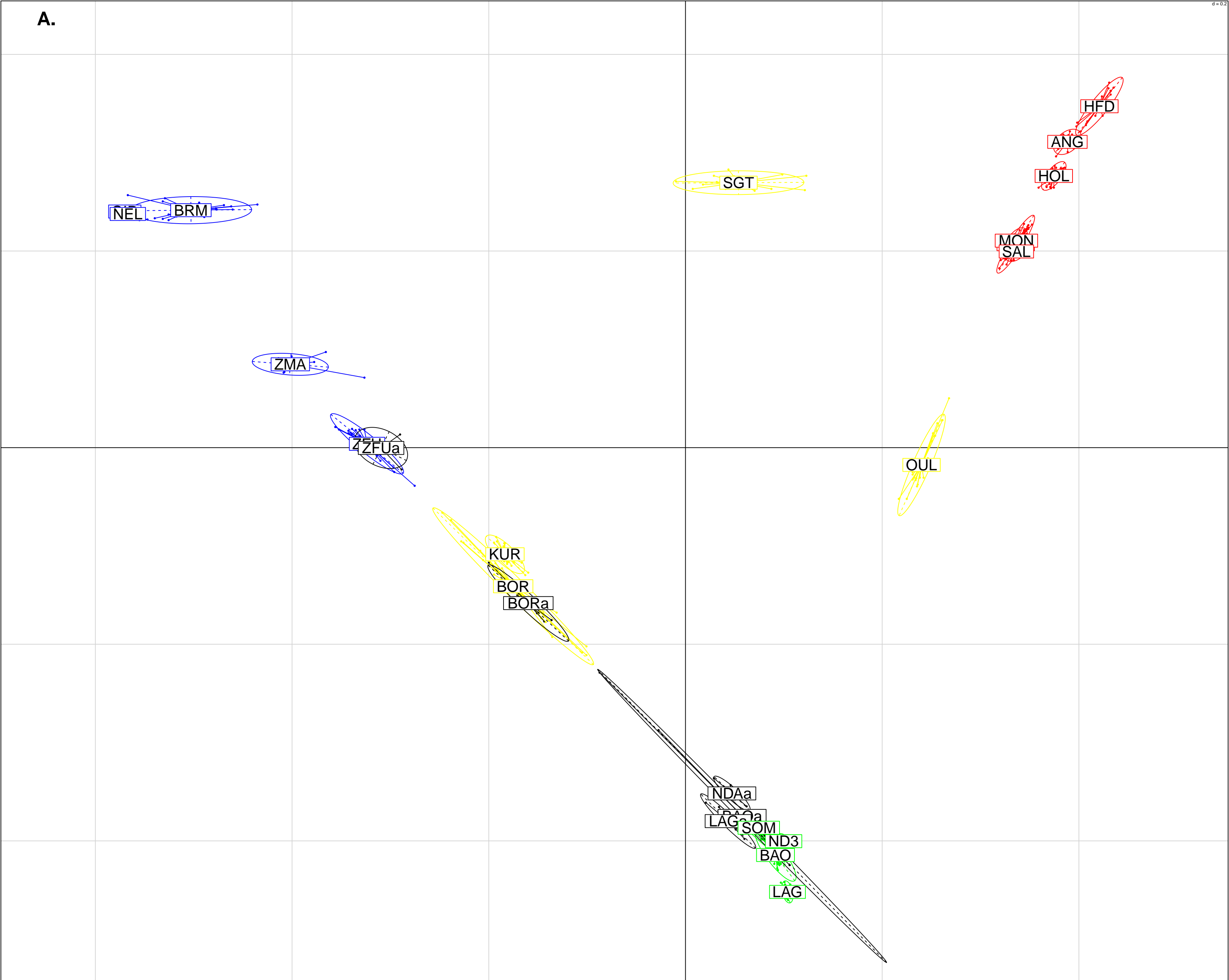

**B.**

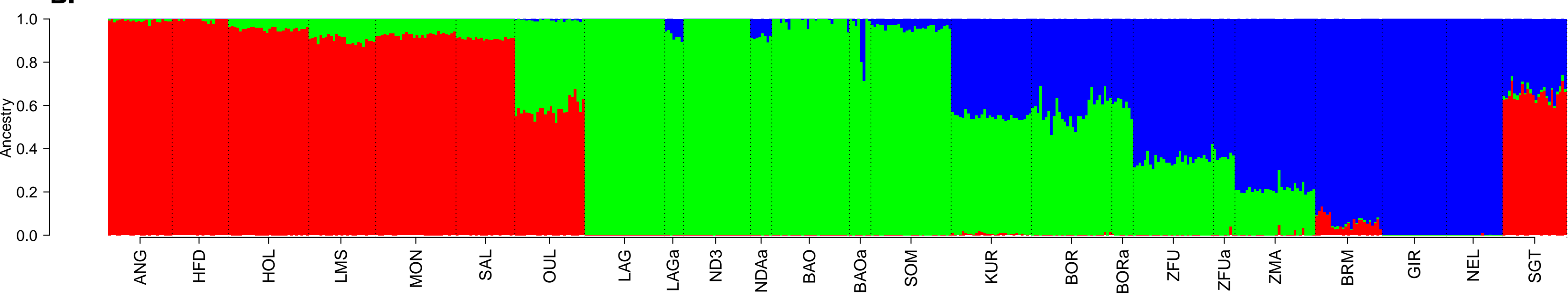

Supplement: S1 Fig — A. PCA results. The individuals are plotted on the first two principal components according to their coordinates. Ellipses characterize the dispersion of each breed around its centre of gravity. The EUT, AFT and ZEB breeds are plotted in red, green and blue, respectively. The EUTxAFT, EUTxZEB and AFTxZEB admixed breeds previously sampled are shown in yellow and the newly sampled populations (BAOa, BORa, LAGa, NDAa and ZFUa) in black. B. Unsupervised hierarchical clustering results for the 545 individuals genotyped for 43,845 SNPs with an inferred number of clusters k = 3 obtained with Admixture 1.04. For each individual, the proportions of each cluster (y axis) which were interpreted as representative of EUT, AFT and ZEB ancestries are plotted in red, green and blue, respectively. (PDF) [file pone.0126498.s001.pdf]
